# Colonization of the gut by *Klebsiella pneumoniae* and its multidrug-resistant strains is well marked in preterm neonates

**DOI:** 10.3389/fcimb.2026.1762624

**Published:** 2026-04-29

**Authors:** Eman Mohammed El-Shanqetti, Hibah M. Albasri, Moayad S. Waznah, Fatthy Mohamed Morsy

**Affiliations:** 1 Department of Biology, College of Science, Taibah University, Madinah, Saudi Arabia; 2 Bacteriology Section, Botany and Microbiology Department, Faculty of Science, Assiut University, Assiut, Egypt

**Keywords:** Carbapenem-resistant enterobacteria, *Escherichia coli*, *Klebsiella pneumoniae*, multidrug-resistant bacteria, preterm neonates

## Abstract

**Background:**

The colonization of preterm neonates by multidrug-resistant Enterobacteriaceae, especially in neonatal intensive care units (NICUs), remains poorly understood despite its potential role in healthcare-associated transmission. This study addresses this gap by characterizing gut colonization by Enterobacteriaceae, focusing on carbapenem-resistant *Klebsiella pneumoniae* in preterm neonates.

**Results:**

Samples of preterm neonates’ stool were cultured on eosin methylene blue (EMB) agar, followed by biochemical identification, 16S rRNA gene sequencing, and antibiotic susceptibility testing using the VITEK 2 system. All preterm neonates aged 2 weeks or older exhibited colonization by enterobacteria. *K. pneumoniae* was the most prevalent species, identified in 56.2% (9/16) of samples, with *K. quasipneumoniae* present in an additional 6.3% (1/16). Two *K. pneumoniae* isolates were multidrug-resistant; one exhibited carbapenem resistance, and one showed intermediate susceptibility. These strains were also resistant to multiple other antibiotics. *Escherichia coli* was detected in 50% (8/16) of cases but exhibited no carbapenem resistance.

**Conclusions:**

This study demonstrates that preterm neonates in NICUs can harbor multidrug-resistant *K. pneumoniae* in their gut, highlighting the gut as a potential reservoir and source of CRE (carbapenem-resistant Enterobacteriaceae) dissemination. These findings stress the need for enhanced surveillance, strict infection control practices, and strategies to prevent gut colonization and fecal–oral transmission of multidrug-resistant bacteria in vulnerable neonatal populations. All strains of *K. pneumoniae*, including those resistant or with intermediate resistance to carbapenems, displayed widespread sensitivity to gentamicin (GM) and tigecycline (TGC) *in vitro*, suggesting potential clinical utility, though further evaluation is warranted.

## 1 Introduction

Multidrug-resistant bacteria pose a growing challenge in combating their healthcare-associated infections (HAIs), where only a limited range of antibiotics is available for effective treatment. Carbapenems, the most recently developed β-lactams, such as meropenem and imipenem (currently marketed as imipenem/cilastatin), have a broad spectrum of antibacterial activity and are generally used as a last resort in the treatment of multidrug-resistant bacterial infections (Nordmann et al., 2012a; Ellappan et al., 2018; Lee and Bradley, 2019; Elshamy and Aboshanab, 2020; Mohamed et al., 2021). In healthcare settings, where multidrug-resistant Gram-negative bacteria are already endemic, the spread of carbapenem-resistant bacterial strains is particularly problematic (Pitout et al., 2015). Carbapenem resistance in Enterobacteriaceae, particularly *K. pneumoniae*, arises through multiple mechanisms that often act synergistically. The most prominent mechanism involves the production of carbapenemases, including KPC (*K. pneumoniae* carbapenemase), NDM (New Delhi metallo-β-lactamase), VIM (Verona integron-encoded metallo-β-lactamase), and OXA-48-like enzymes, which hydrolyze carbapenems and render them ineffective (Nordmann et al., 2012b; Logan and Weinstein, 2017). Additional resistance is mediated through porin loss or mutation (e.g., OmpK35 and OmpK36), which decreases antibiotic uptake and enhances the effect of β-lactamase activity (Wang et al., 2020). Overexpression of efflux pumps, such as AcrAB-TolC, also plays a role by actively exporting antibiotics out of the bacterial cell (Nishino et al., 2021). In clinical isolates, these mechanisms frequently coexist, contributing to high-level and broad-spectrum antibiotic resistance. Furthermore, the recent emergence of hypervirulent carbapenem-resistant *K. pneumoniae* (CR-hvKp), which combines antimicrobial resistance with enhanced virulence factors, has intensified the threat of hospital-acquired infections, particularly in NICUs (Gu et al., 2018; Shankar et al., 2022).

In addition to carbapenems, colistin (polymyxin E) is widely regarded as a last-resort antibiotic for treating infections caused by carbapenem-resistant and multidrug-resistant Gram-negative bacteria, including *Klebsiella pneumoniae* and *Escherichia coli*. Colistin has traditionally been reserved for critically ill patients when other treatment options fail; however, the emergence and spread of colistin-resistant strains, including plasmid-mediated resistance associated with *mcr* genes, pose a serious global public health threat by further limiting the therapeutic options. Recent meta-analyses and surveillance data have documented significant rates of colistin resistance among Enterobacteriaceae, particularly among carbapenem-resistant isolates, underscoring the pressing need for enhanced antimicrobial stewardship and continuous monitoring of resistance trends (Mondal et al., 2024).

Healthcare-associated infections by multidrug-resistant bacteria are posing a huge burden on healthcare systems worldwide and are associated with high morbidity and mortality (Sousa et al., 2021; Coque et al., 2023; Freitas and Werner, 2023; Duan et al., 2024; Valzano et al., 2024). Plumbing systems, components, and sanitary installations in hospital restrooms may serve as reservoirs and potential incubation sites for multidrug-resistant bacteria (Hocquet et al., 2016; Kizny Gordon et al., 2017; Park et al., 2020; Valzano et al., 2024). Several factors increase the risk of healthcare-associated infections, including the overuse of broad-spectrum antibiotics, lack of sterility or improper sterilization of invasive devices, the intensive care unit (ICU) environment, and other contributing factors. The persistence and continuous circulation of multidrug-resistant bacteria in these settings represent a significant risk, particularly in ICUs. One possible incubation site for multidrug-resistant bacterial strains is the colonization of preterm newborns’ guts by these strains. To investigate whether preterm neonates’ guts serve as a reservoir for multidrug-resistant enterobacteria, this study examined the presence of both normal resident enterobacteria and multidrug-resistant strains in their stool samples.

## 2 Materials and methods

The colonization of preterm neonates’ gut by normal resident enterobacteria and their multidrug-resistant strains was investigated by exploring their occurrence in preterm neonates’ stool.

### 2.1 Ethics approval and consent to participate

This study was conducted according to approval from the ethics committee of the Institutional Review Board (IRB), General Directorate of Health Affairs in Madinah, Kingdom of Saudi Arabia (KSA) [IRB Committee Head, Dr. Yasmeen Talal AlJehani]. Informed consents were obtained from parents of preterm neonates enrolled in the study for the collection of the preterm neonates’ stool. All methods were performed in accordance with the relevant guidelines and regulations of approval from the ethics committee of the IRB. This study was conducted in compliance with the Helsinki Declaration (https://eur01.safelinks.protection.outlook.com/?url=https%3A%2F%2Fwww.wma.net%2Fpolicies-post%2Fwma-declaration-of-helsinki%2F&data=05%7C02%7Cfmorsy%40aun.edu.eg%7C2aafbc6d2c1443190c6f08dd9723361c%7C794544284dbe401ab2ab64a5a7069e2b%7C1%7C0%7C638832899673025729%7CUnknown%7CTWFpbGZsb3d8eyJFbXB0eU1hcGkiOnRydWUsIlYiOiIwLjAuMDAwMCIsIlAiOiJXaW4zMiIsIkFOIjoiTWFpbCIsIldUIjoyfQ%3D%3D%7C0%7C%7C%7C&sdata=BcL2viEy1ud9PK7PfP9LH5B6Duj6MOwE0rFhVqeC3IA%3D&reserved=0).

Stool samples from preterm neonates were collected from the NICUs of three hospitals in Madinah, KSA (cases 1, 2, and 3 were collected from Maternity and Children Hospital, cases 4, 5, and 6 from Saudi German Hospital Madinah, and cases 7 to 21 from Ohud Hospital). The different preterm neonates were chosen randomly to obtain stool samples for a broad view of the study. All preterm neonates present in the NICUs of the three hospitals between September 28, 2021 and November 14, 2021 were included in the study without applying any selection criteria. A longitudinal follow-up was carried out for certain preterm neonates in the NICUs whenever feasible and was documented as visit 1 and visit 2. All details of the preterm neonate subjects, including age and other details such as antibiotic treatments received, are outlined in **Table 1**, where all investigated preterm neonate cases aged 2 weeks or older equal to 16 cases. Once the samples of preterm neonate stool, normally obtained in diapers, were collected, they were sent for microbiological analysis in the bacteriology laboratory at Taibah University and used directly to explore the occurrence of enterobacteria and its multidrug-resistant strains. The occurrence of enterobacteria in the stool of preterm neonates was monitored using eosin methylene blue (EMB) agar. No antibiotics were added to the EMB agar or any other culture medium in this study.

**Table 1:** Colonization of the gut of preterm neonates by *K. pneumoniae*, *E. coli*, and other bacteria following intravenous antibiotic treatment.

Sampling Time	Preterm neonates, subject #	Age, days	Gender	Gestational age (weeks)	Diagnostics	Types of antibiotics treated with	CFU/gm stool	Bacterial strains colonizing preterm neonates’ gut	Accession number
1st week	Case 3	2	Male	31	Suspected sepsis	Ampicillin 50 mg/iv/q12h Cefotaxime 50 mg/iv/q12/h	–	ND	–
	Case 4 (visit 1)	5	Male	32	Suspected sepsis	Gentamicin–ampicillin 50 mg/iv/q12h	–	ND	–
	Case 5 (visit 1)	6	Female	32	Suspected sepsis	Gentamicin–ampicillin 50 mg/iv/q12h	–	ND	–
	Case 9	2	Male	30	Suspected sepsis	Meropenem 80 mg/iv/q12h Cefotaxime 80 mg/iv/q12h	–	ND	–
	Case 18	3	Male	33	Suspected sepsis	Ampicillin 90 mg/iv/q12h Gentamicin 8 mg/iv/q36h	–	ND	–
	Case 19	5	Male	26	Suspected sepsis	Ampicillin 12 mg/iv/q12h Cefotaxime 50 mg/iv/q48h	–	ND	–
>1 week	Case 6 (visit 1)	8	Female	28	Suspected sepsis	Ampicillin 50 mg/iv/q12h Cefotaxime 50 mg/iv/q12h	–	ND	–
	Case 1	22	Male	27	Suspected sepsis	Vancomycin 10 mg/iv/q36h	1.134 × 10^8^	*Escherichia coli* EFTU1	PP156718
	Case 2	18	Female	25	Suspected sepsis	Ampicillin 50 mg/iv/q12h Cefotaxime 50 mg/iv/q12/h Fluconazole 13 mg/iv/q12h	306.18 × 10^6^	*Klebsiella pneumoniae* EFTU101	PP157088
	Case 4 (visit 2)	15	Male	32	Suspected sepsis	Stopped antibiotics before 5 days	453.6 × 10^4^	*Escherichia coli* EFTU2	PP157624
	Case 5 (visit 2)	16	Female	33	Suspected sepsis	Gentamicin–ampicillin 50 mg/iv/q12h	396.9 × 10^10^	*Klebsiella pneumoniae* EFTU102	PP177257
	Case 6 (visit 2)	18	Female	28	Suspected sepsis	Ampicillin 50 mg/iv/q12h Cefotaxime 50 mg/iv/q12h	742 × 10^10^	*Klebsiella pneumoniae* EFTU103	PP177305
	Case 7	44	Female	26	Suspected sepsis	Vancomycin 10 mg/iv/q12h Meropenem 20 mg/iv/q12h	204.1 × 10^4^	*Klebsiella pneumoniae* EFTU104	PP177307
	Case 8	44	Female	26	Suspected sepsis	Vancomycin 10 mg/iv/q12h Meropenem 20 mg/iv/q12h	1.3608 × 10^8^	*Klebsiella pneumoniae* EFTU105	PP177367
	Case 10	44	Male	29	Suspected sepsis	Vancomycin 15 mg/iv/q12 h Stopped antibiotics before 18 days	793.8 × 10^4^	*Klebsiella pneumoniae* EFTU3	PP157908
	Case 11	24	Female	31	Suspected sepsis	No antibiotic	136.1 × 10^4^	*Escherichia coli* EFTU4	PP182268
	Case 13	21	Female	25	Suspected sepsis	Meropenem 27 mg/iv/q12h Colistin 45 mg/iv/q12h	147.4 × 10^4^	*Escherichia coli* EFTU5	PP187030
	Case 14	16	Male	33	Suspected sepsis	Gentamicin 8 mg/iv/q36h Ampicillin 76 mg/iv/q12h	113.4 × 10^4^	*Klebsiella pneumoniae* EFTU110	PP177465
	Case 15	46	Male	27	Suspected sepsis	Vancomycin 10 mg/iv/q8h Meropenem 20 mg/iv/q8h	4.625 × 10^7^	*Escherichia coli* EFTU6	PP187981
								*Klebsiella pneumoniae* EFTU113	PP204007
								*Acinetobacter baumannii* EFTU111	PP177550
	Case 16	29	Male	29	Suspected sepsis	Ampicillin 65 mg/iv/q12h Gentamicin 65 mg/iv/q48h	181.4 × 10^4^	*Acinetobacter baumannii* EFTU114	PP177613
	Case 17	32	Female	29	Suspected sepsis	Ampicillin 60 mg/iv/q12h Cefotaxime 60 mg/iv/q48h	1.9958 × 10^8^	*Escherichia coli* EFTU7	PP188038
								*Klebsiella quasipneumoniae* EFTU115	PP177908
	Case 20	64	Male	31	Suspected sepsis	Meropenem 50 mg/iv/q8h Colistin 50 mg/iv/q8h	3.8 × 10^6^	*Escherichia coli* EFTU8	PP188042
								*Klebsiella pneumoniae* EFTU116	PP177909
	Case 21	14	Female	31	Suspected sepsis	Ampicillin 67 mg/iv/q12h Gentamicin 67 mg/iv/q36h Stopped antibiotics before 6 days	63504 × 10^3^	*Escherichia coli* EFTU9	PP188096

ND, not detected.

An approximately 1 g portion of each stool sample was aseptically suspended in 9 mL of sterile normal saline to prepare an initial dilution. Serial dilutions were subsequently performed, and aliquots were inoculated onto EMB agar, followed by incubation at 37°C for 24 h. Colonies showing characteristic morphology, such as metallic-green-sheen colonies suggestive o*f Escherichia coli* or large, dome-shaped, pink to purple mucoid colonies suggestive of *Klebsiella* spp., were identified. Colony-forming units (CFU) were calculated, and bacterial load was further estimated using the most probable number (MPN) technique (MacFaddin, 1985). Plates with countable colonies were used to determine the CFU of dominant morphotypes. Representative colonies were subcultured on EMB agar to obtain pure isolates. The isolates were further purified on Luria–Bertani (LB) agar, cultured in LB broth at 37°C for 24 h, and preserved in LB broth containing 20% glycerol at −80°C. Morphological and biochemical characterizations of enterobacteria isolates were conducted for identification as outlined in Bergey’s manual (Brenner et al., 2005) and confirmed using VITEK 2 technology. Further genetic identification of bacterial strains was performed through molecular analysis based on 16S rRNA gene sequencing and phylogenetic analysis. Antimicrobial susceptibility testing of all isolated bacterial strains was carried out using the VITEK^®^ 2 COMPACT automated susceptibility testing system (bioMérieux, Marcy-l’Étoile, France) at Al Madinah Al Munawarah Hospital, King Salman Bin Abdulaziz Medical City. The susceptibility profiles of the isolates against various antibiotics were determined by measuring the minimum inhibitory concentrations (MICs) generated by the VITEK 2 system and interpreted according to Clinical and Laboratory Standards Institute (CLSI, 2023) guidelines. To ensure the accuracy and reliability of antimicrobial susceptibility testing, reference quality control strains *Escherichia coli* ATCC 25922, *Klebsiella pneumoniae* ATCC 700603, and *Acinetobacter baumannii* NCTC 13304 were included and tested in parallel according to standard laboratory practices, and the results were validated within acceptable CLSI quality control ranges. The antimicrobial agents evaluated included ampicillin/sulbactam (AMP), amoxicillin/clavulanic acid (AMOX), piperacillin/tazobactam (TZP), cefalotin (CF), cefoxitin (FOX), ceftazidime (CAZ), ceftriaxone (CRO), cefepime (FEP), imipenem (IMI), meropenem (MERO), amikacin (AK), gentamicin (GM), ciprofloxacin (CIP), tigecycline (TGC), nitrofurantoin (FT), and trimethoprim/sulfamethoxazole (SXT).

### 2.2 Statistical analysis

The association between the hospital and the distribution of bacterial strains was evaluated using the chi-square test of independence. Degrees of freedom were calculated based on the number of bacterial categories and the number of hospitals included in the analysis. A *p*-value <0.05 was considered statistically significant. All analyses were performed using standard statistical software.

### 2.3 Genetic identification of bacterial strains by phylogenetic analysis of 16S rRNA-encoding gene sequence

The genetic identification of bacterial strains using molecular biological technology was conducted through a phylogenetic analysis of the 16S rRNA-encoding gene sequence of each bacterial strain.

#### 2.3.1 PCR amplification of the 16S rRNA gene

Extraction and purification of genomic DNA from bacterial cultures were conducted using Promega Wizard (Promega Corporation, Madison, WI, USA) genomic DNA purification kit according to the instructions described by the manufacturer. The purified genomic DNA was used as a template to amplify a nearly full-length nucleotide sequence of the 16S rRNA-encoding gene by polymerase chain reaction (PCR). The universal forward (27F, 5′-AGAGTTTGATC[A/C]TGGCTCAG-3′) and reverse (1492R, 5′-G[C/T]TACCTTGTTACGACTT-3′) primers were used for the PCR amplification of the nearly full-length sequence of the 16S rRNA-encoding gene (Lane, 1991). The PCR amplification of the nearly full-length sequence of the 16S rRNA-encoding gene was conducted in a thermal cycler (model 2720; Applied Biosystems, Foster City, CA, USA) using a 25-µL reaction mixture (25 µL) with the following composition: 2.5 µL 10× Taq buffer (100 mM Tris-HCl, pH 8), 100 mM deoxynucleoside triphosphates (dNTPs) (Invitrogen, Carlsbad, CA, USA), 1.25 mM MgCl_2_, 1.2 mM forward and reverse primers, 0.5 U Taq DNA polymerase (Invitrogen, USA), and approximately 5 ng of the template bacterial genomic DNA. The following PCR program was used for the amplification: initial denaturation at 95 °C for 5 min, followed by 35 cycles of amplification [94 °C (denaturation) for 1 min, 56 °C (annealing) for 1 min, and 72 °C (extension) for 1 min], with a final extension of 10 min at 72 °C. Analysis of the PCR amplification products was conducted using agarose gel electrophoresis using 1% agarose gels containing 5 µg/mL ethidium bromide compared to a DNA-size marker (1 kb Plus DNA ladder; Invitrogen, USA).

#### 2.3.2 Nucleotide sequence analysis

Purification and cycle sequencing of the PCR products were conducted at the Macrogen Korea sequencing facility located in Seoul, South Korea. The purified PCR products were subjected to direct cycle sequencing at the Macrogen Korea sequencing facility by using the automated fluorescent dye terminator sequencing method (Sanger et al., 1977) in the 3730XL DNA analyzer (Applied Biosystems, CA, USA) using the same forward (27F) and reverse (1492R) primers for sequencing in both directions. Using the National Center for Biotechnology and Information (NCBI) server’s nucleotide–nucleotide BLAST search tool, the sequence reads of the 16S rRNA-encoding gene of each isolated bacterial strain colonizing the preterm neonates’ gut were assembled and compared with the closest matches of bacterial strains recorded in the GenBank at www.ncbi.nlm.nih.gov/blast/Blast.cgi. Alignments of sequences of the 16S rRNA gene were conducted using ClustalW1.83 XP (Thompson et al., 1997). Using the neighbor-joining method (Saitou and Nei, 1987), the derived phylogenetic tree of the 16S rRNA gene sequences was constructed with MEGA11 software (Tamura et al., 2021). *Bacillus subtilis* strain JCM 1465 (accession number NR_113265) was used as an outgroup.

## 3 Results

In this study, the colonization of the preterm neonates’ gut by enterobacteria and its multidrug-resistant strains was investigated to explore whether the gut of preterm neonates *per se* could be the source of dissemination of these multidrug-resistant strains and their site of incubation. This was conducted by following the occurrence of enterobacteria in the stool of preterm neonates (**Table 1**) on EMB agar, followed by morphological and biochemical characterizations for identification and by using VITEK 2 technology. Testing of antibiotic susceptibility using VITEK 2 technology automated susceptibility testing system for all isolated bacterial strains was also conducted (**Supplementary Tables S1**, **S2**). The gestational age of all investigated preterm neonate cases ranged between 25 and 33 weeks (**Table 1**).

The chi-square test of independence yielded a chi-square statistic of 0.56 with four degrees of freedom and an associated *p*-value of approximately 0.966. These results indicate no statistically significant association between the hospital and the distribution of bacterial strains among colonized cases. Thus, the pattern of bacterial colonization appeared to be consistent across the participating hospitals. The absence of hospital-specific clustering suggests that colonization is not confined to a single ward or institution but reflects a widespread risk across neonatal intensive care units.

All investigated preterm neonate cases were receiving antibiotic treatments intravenously in the intensive care units as outlined in **Table 1**. The ages of the investigated randomly selected preterm neonate cases ranged between 2 and 64 days (**Table 1**).

Colonization was strongly age dependent: all neonates ≤8 days were negative, whereas colonization was universal among those ≥2 weeks, indicating that early life (first week) represents a critical window where the gut remains uncolonized. The bacterial load varied considerably, from 10^4^ to >10^10^ CFU/g stool, indicating both low-level and heavy colonization in different neonates. No clear gender-based differences in colonization patterns were observed, as both male and female neonates were colonized by similar bacterial species. All colonized neonates were diagnosed with suspected sepsis, reinforcing the clinical overlap between gut colonization and neonatal infection risk (**Table 1**).

**Figure 1:**
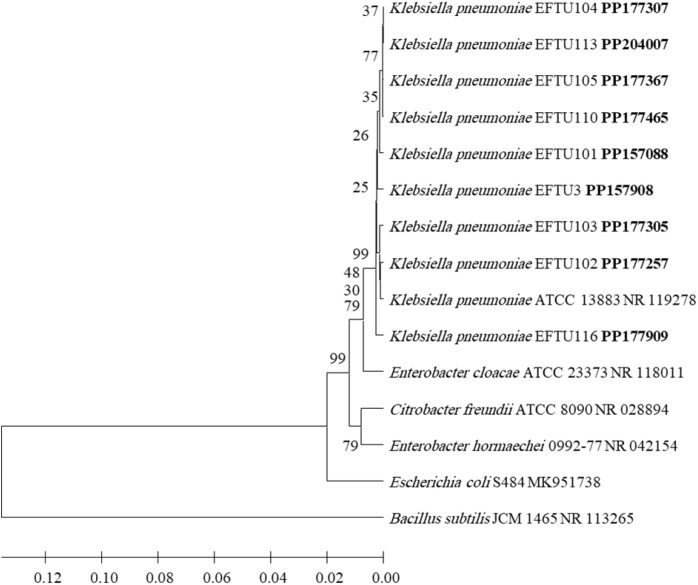
Phylogenetic analysis of 16S rRNA-encoding gene of *K. pneumoniae* strains isolated from preterm neonates’ stool. The isolated *K. pneumoniae* strains’ phylogenetic tree shows the relationship with the closest neighbor strains of bacteria from NCBI. The accession numbers of the 16S rRNA-encoding genes of isolated *K. pneumoniae* strains are shown in boldface. The isolated *K. pneumoniae* strains’ and other bacterial strains’ neighbor-joining trees were determined using the nearly full-length gene sequences of 16S rDNA and the frequency filter in the analysis package of MEGA 11 software. An outgroup, *Bacillus subtilis* JCM 1465 (accession number NR_113265), was used in the analysis. The scale bar shown indicates a 2% estimated difference in sequence. The NCBI database accession numbers and strain names are displayed in the phylogenetic tree and are also listed here: *K. pneumoniae* EFTU104, PP177307; *K. pneumoniae* EFTU113, PP204007; *K. pneumoniae* EFTU105, PP177367; *K. pneumoniae* EFTU110, PP177465; *K. pneumoniae* EFTU101, PP157088; *K. pneumoniae* EFTU3, PP157908; *K. pneumoniae* EFTU103, PP177305; *K. pneumoniae* EFTU102, PP177257; *K. pneumoniae* ATCC 13883, NR_119278; *K. pneumoniae* EFTU116, PP177909; *Enterobacter cloacae* ATCC 23373, NR_118011; *Citrobacter freundii* ATCC 8090, NR_028894; *Enterobacter hormaechei* 0992-77, NR_042154; *Escherichia coli* S484, MK951738; *Bacillus subtilis* JCM 1465, NR_113265. Bootstrap values are displayed on the clades of the phylogenetic tree.

**Figure 2:**
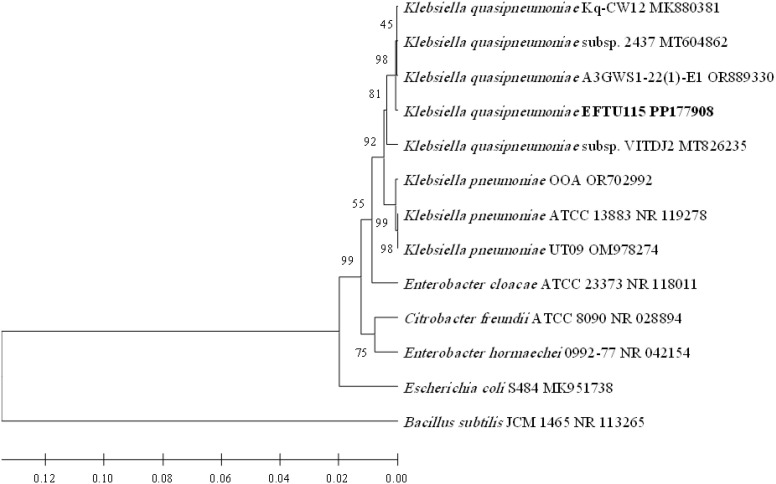
Phylogenetic analysis of 16S rRNA-encoding gene of *K. quasipneumoniae* strain EFTU115 isolated from preterm neonates’ stool. The isolated *K. quasipneumoniae* strain EFTU115 phylogenetic tree shows the relationship with the closest neighbor strains of bacteria from NCBI. The accession numbers of the 16S rRNA-encoding genes of isolated *K. quasipneumoniae* strain EFTU115 are shown in boldface. The isolated *K. quasipneumoniae* strain EFTU115’s and other bacterial strains’ neighbor-joining trees were determined using the nearly full-length gene sequences of 16S rDNA and the frequency filter in the analysis package of MEGA 11 software. An outgroup, *Bacillus subtilis* JCM 1465 (accession number NR_113265), was used in the analysis. The scale bar shown indicates a 2% estimated difference in sequence. The NCBI database accession numbers and strain names are displayed in the phylogenetic tree and are also listed here: *K. quasipneumoniae* Kq-CW12, MK880381; *K. quasipneumoniae*. 2437, MT604862; *K. quasipneumoniae* A3GWS1-22(1)-E1, OR889330; *K. quasipneumoniae* EFTU115, PP177908; *K. quasipneumoniae* VITDJ2, MT826235; *K. pneumoniae* OOA, OR702992; *K. pneumoniae* ATCC 13883, NR_119278; *K. pneumoniae* UT09, OM978274; *Enterobacter cloacae* ATCC 23373, NR_118011; *Citrobacter freundii* ATCC 8090, NR_028894; *Enterobacter hormaechei* 0992-77, NR_042154; *Escherichia coli* S484, MK951738; *Bacillus subtilis* JCM 1465, NR_113265. Bootstrap values are displayed on the clades of the phylogenetic tree.

**Figure 3:**
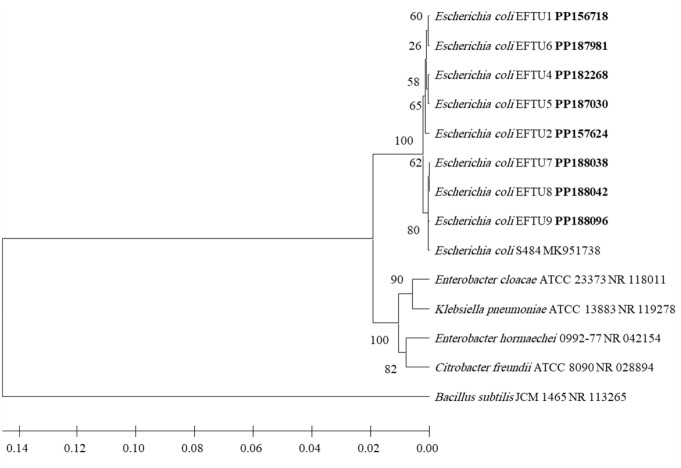
Phylogenetic analysis of 16S rRNA-encoding gene of *Escherichia coli* strains isolated from preterm neonates’ stool. The isolated *Escherichia coli* strains’ phylogenetic tree shows the relationship with the closest neighbor strains of bacteria from NCBI. The accession numbers of the 16S rRNA-encoding genes of isolated *Escherichia coli* strains are shown in boldface. The isolated *Escherichia coli* strains’ and other bacterial strains’ neighbor-joining trees were determined using the nearly full-length gene sequences of 16S rDNA and the frequency filter in the analysis package of MEGA 11 software. An outgroup, *Bacillus subtilis* JCM 1465 (accession number NR_113265), was used in the analysis. The scale bar shown indicates a 2% estimated difference in sequence. The NCBI database accession numbers and strain names are displayed in the phylogenetic tree and are also listed here: *Escherichia coli* EFTU1, PP156718; *Escherichia coli* EFTU6, PP187981; *Escherichia coli* EFTU4, PP182268; *Escherichia coli* EFTU5, PP187030; *Escherichia coli* EFTU2, PP157624; *Escherichia coli* EFTU7, PP188038; *Escherichia coli* EFTU8, PP188042; *Escherichia coli* EFTU9, PP188096; *Escherichia coli* S484, MK951738; *Enterobacter cloacae* ATCC 23373, NR_118011; *K. pneumoniae* ATCC13883, NR_119278; *Enterobacter hormaechei* 0992-77, NR_042154; *Citrobacter freundii* ATCC 8090, NR_028894; *Bacillus subtilis* JCM 1465, NR_113265. Bootstrap values are displayed on the clades of the phylogenetic tree.

**Figure 4:**
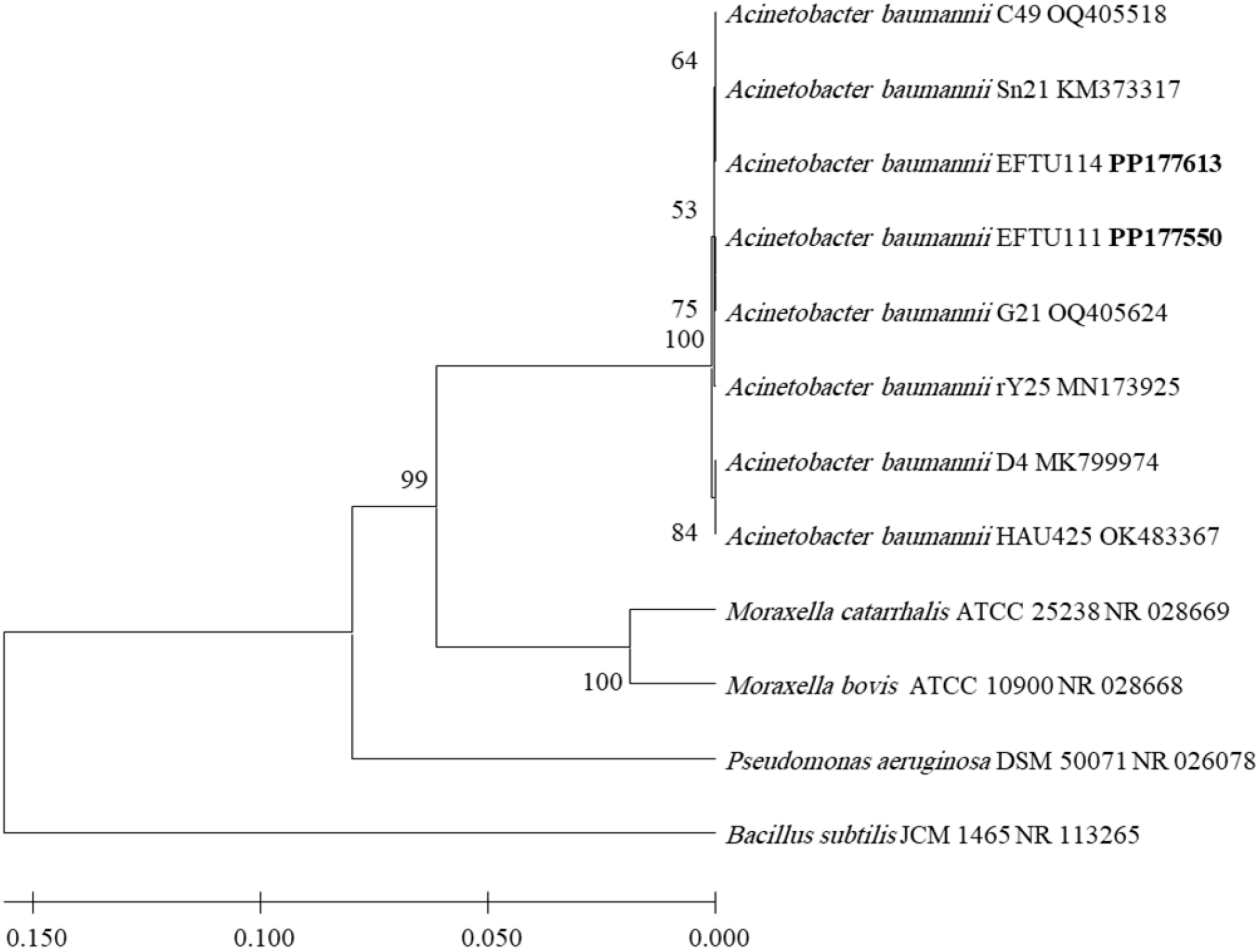
Phylogenetic analysis of 16S rRNA-encoding gene of *Acinetobacter baumannii* (a non-Enterobacteriaceae bacterium) strains isolated from preterm neonates’ stool. The isolated *Acinetobacter baumannii* strains’ phylogenetic tree shows the relationship with the closest neighbor strains of bacteria from NCBI. The accession numbers of the 16S rRNA-encoding genes of isolated *Acinetobacter baumannii* strains are shown in boldface. The isolated *Acinetobacter baumannii* strains’ and other bacterial strains’ neighbor-joining trees were determined using the nearly full-length gene sequences of 16S rDNA and the frequency filter in the analysis package of MEGA 11 software. An outgroup, *Bacillus subtilis* JCM 1465 (accession number NR_113265), was used in the analysis. The scale bar shown indicates a 2% estimated difference in sequence. The NCBI database accession numbers and strain names are displayed in the phylogenetic tree and are also listed here: *Acinetobacter baumannii* C49, OQ405518; *Acinetobacter baumannii* Sn21, KM373317; *Acinetobacter baumannii* EFTU114, PP177613; *Acinetobacter baumannii* EFTU111, PP177550; *Acinetobacter baumannii* G21, OQ405624; *Acinetobacter baumannii* rY25, MN173925; *Acinetobacter baumannii* D4, MK799974; *Acinetobacter baumannii* HAU425, OK483367; *Moraxella catarrhalis* Ne 11 ATCC 25238, NR_028669; *Moraxella bovis* L-3 ATCC 10900, NR_028668; *Pseudomonas aeruginosa* DSM 50071, NR_026078; *Bacillus subtilis* JCM 1465, NR_113265. Bootstrap values are displayed on the clades of the phylogenetic tree.

After identification by morphological and biochemical characterizations and by using VITEK 2 technology, the molecular biological identification of all isolated strains of bacteria was also performed by the phylogenetic analysis of each bacterial strain 16S rRNA-encoding gene sequence (**Figures 1**–**4**). Analyzing the PCR amplification products on agarose gel electrophoresis showed 1.5-kb bands that match the expected amplification size (1,500 bp) using the universal forward 27F and reverse 1492R primers, indicating a successful amplification of the nearly full-length bacterial 16S rRNA gene. The nearly full-length nucleotide sequence of the 16S rRNA-encoding gene of all isolated bacterial strains deposited in NCBI’s GenBank nucleotide sequence database was used for the phylogenetic-analysis-based identification of these bacterial strains in comparison to the closest matches of bacterial strains recorded in GenBank (**Figures 1**–**4**).

*Klebsiella pneumoniae* (**Figure 1**) was found to be colonizing the gut in nine cases of preterm neonates in neonatal intensive care units (**Table 1**), representing 56.2% of the total colonized cases (16 cases). The relevant bacterium *K. quasipneumoniae* (**Figure 2**) was found in one case, representing 6.3%. Thus, both bacteria represent a total of 62.5% of the total colonized cases. Some cases contained more than one genus (**Table 1**), where *E. coli* (**Figure 3**) was found colonizing the gut in eight cases, representing 50% of the total colonized cases of preterm neonates (16 cases) in neonatal intensive care units (**Table 1**).

To explore the occurrence of multidrug-resistant enterobacteria, the isolated bacterial strains’ susceptibility to various antibiotics and the resistance versus sensitivity of these bacterial strains were assessed from the MIC (**Supplementary Tables S1**, **S2**), followed by using the VITEK.2 device. Strains of *K. pneumoniae* (**Figure 1**) that colonized the gut of the investigated preterm neonates and showing extensive multidrug resistance was clear in two strains (**Supplementary Table 1**). One strain *K. pneumoniae* EFTU110 was resistant to carbapenems (meropenem and imipenem) and another *K. pneumoniae* EFTU113 showed intermediate resistance to these antibiotics. These two strains of *K. pneumoniae* demonstrated resistance to most other tested antibiotics (**Supplementary Table 1**). The occurrence of carbapenem-resistant *K. pneumoniae* in the stool of preterm neonates (**Supplementary Table 1**) highlights the ability of these multidrug-resistant bacterial strains to colonize the gut of preterm neonates, where their incubation site is located, allowing them to spread in neonatal intensive care units. In contrast to *K. pneumoniae*, none of the *E. coli* strains (**Figure 3**) colonizing the gut of the investigated preterm neonates’ cases in this study showed resistance to the carbapenems tested, meropenem and imipenem (**Supplementary Table 2**).

Extended-spectrum beta-lactamase (ESBL)-producing bacterial strains were detected in this study in four cases in *K. pneumoniae* and in *E. coli* (**Supplementary Tables S1**, **S2**). While both *K. pneumoniae* and *E. coli* showed ESBL production, only *K. pneumoniae* exhibited carbapenem resistance, reinforcing its role as the primary driver of multidrug resistance in this cohort.

Along with enterobacteria, the non-Enterobacteriaceae *Acinetobacter baumannii* known for its active healthcare-associated infections was found in the stool of two cases, representing 12.5% of the total colonized preterm neonate cases (**Table 1**), as confirmed by identification using the phylogenetic analysis of the 16S rRNA-encoding gene sequence (**Figure 4**). *A. baumannii* strains colonizing the gut of preterm neonates, isolated from the two cases, did not show resistance to the tested carbapenems, meropenem and imipenem (**Supplementary Table 1**); thus, the carbapenems multidrug-resistant strains were found only among *K. pneumoniae.*

All strains of *K. pneumoniae* and 87.5% (seven out of eight) of *E. coli* strains exhibited resistance to ampicillin/sulbactam (**Table 2**), suggesting significant resistance in both *K. pneumoniae* and *E. coli* against this antibiotic (**Supplementary Table 1**). The carbapenem-resistant and intermediate multidrug-resistant strains of *K. pneumoniae* (**Supplementary Table 1**) were both susceptible to gentamicin and tigecycline. All other *K. pneumoniae* strains, including the ESBL-producing multidrug-resistant strains, were also found to be susceptible to these two antibiotics (**Supplementary Table 1**). Interestingly, all strains of *E. coli* were also susceptible to these two antibiotics (**Table 2**), including the ESBL-producing multidrug-resistant strains (**Supplementary Table 2**). Additionally, all of the isolated strains of *A. baumannii* and *K. quasipneumoniae* (**Supplementary Table 1**) were also susceptible to gentamicin and tigecycline. These results indicate the possible effectiveness of gentamicin and tigecycline antibiotics in the treatment of multidrug-resistant strains of *K. pneumoniae*.

**Table 2:** Resistant strains of *E. coli* and *K. pneumoniae* colonizing the gut of preterm neonate subjects aged 2 weeks or older to various antibiotics.

	Antibiotics tested	*E. coli*	%	*K. pneumoniae*	%
Strain count		8		9	
ESBLs		4	50	4	44.4
Antibiotics	Ampicillin/sulbactam, AMP	7	87.5	9	100
	Amoxicillin/clavulanic acid, AMOX	0	0	3	33.3
	Piperacillin/tazobactam, TZP	0	0	2	22.2
	Cefalotin, CF	4	50	6	66.6
	Cefoxitin, FOX	0	0	4	44.4
	Ceftazidime, CAZ	4	50	5	55.5
	Ceftriaxone, CRO	4	50	5	55.5
	Cefepime, FEP	4	50	5	55.5
	Imipenem, IMI	0	0	1	11.1
	Meropenem, MERO	0	0	1	11.1
	Amikacin, AK	0	0	1	11.1
	Gentamicin, GM	0	0	0	0
	Ciprofloxacin, CIP	0	0	2	22.2
	Tigecycline, TGC	0	0	0	0
	Nitrofurantoin, FT	0	0	4	44.4
	Trimethoprim/sulfamethoxazole, SXT	1	12.5	5	55.5

ESBLs, extended-spectrum beta-lactamases.

**Figure 5:**
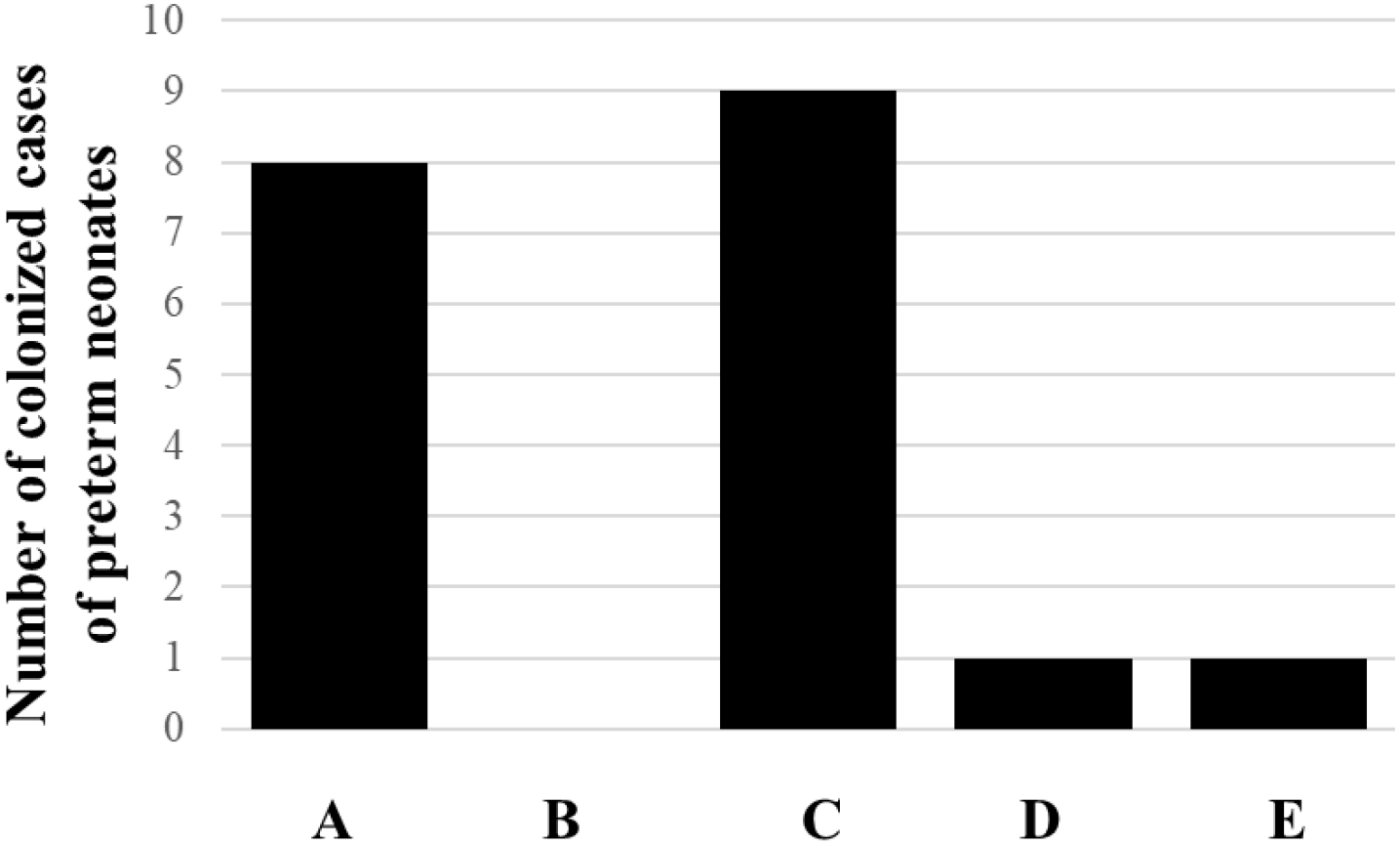
Colonization of preterm neonates’ gut by *E. coli* and *K. pneumoniae.* The number of colonized cases of preterm neonates by *E. coli* [column **(A)**], carbapenems multidrug-resistant *E. coli* [column **(B)**], *K. pneumoniae* [column **(C)**], carbapenems multidrug-resistant *K. pneumoniae* [column **(D)**], and intermediate carbapenems multidrug-resistant *K. pneumoniae* [column **(E)**] is out of the total of 16 preterm neonate subjects aged 2 weeks or older investigated in this study.

The carbapenem-multidrug-resistant *K. pneumoniae* and intermediate-carbapenem-multidrug-resistant *K. pneumoniae* (**Figure 5**) each accounted for 6.3% of the 16 preterm neonate subjects aged 2 weeks or older examined in this study, in contrast to *E. coli* which did not exhibit any carbapenem-multidrug-resistant strains.

Generally, ampicillin/sulbactam resistance was almost universal (87.5% of *E. coli* strains and 100% of *K. pneumoniae* strains), while resistance to last-line carbapenems remained relatively uncommon (22.2% of *K. pneumoniae* strains, including both carbapenem-multidrug-resistant *K. pneumoniae* and intermediate-carbapenem-multidrug-resistant *K. pneumoniae*), reflecting selective resistance pressure in this population. Notably, fluoroquinolone resistance (ciprofloxacin) was detected in 22.2% of *K. pneumoniae* strains despite these drugs not being routinely used in neonates, raising concerns about horizontal gene transfer.

## 4 Discussion

This study was devoted to visualizing whether colonization of the gut of intravenously antibiotic-treated preterm neonates by multidrug-resistant enterobacteria strains constitutes an incubation site in NICUs. The colonization of preterm neonates’ gut by normal resident enterobacteria and multidrug-resistant strains was assessed by analyzing stool samples. While stool samples serve as practical, non-invasive indicators of intestinal bacterial presence, it is important to acknowledge that fecal detection may not fully reflect colonization of the intestinal mucosa. Therefore, the results should be interpreted as evidence of fecal bacterial presence rather than definitive mucosal colonization. All investigated preterm neonates aged 2 to 8 days showed no bacteria in their stool, indicating a delay in colonization, possibly due to antibiotic treatment and the healthcare environment in the NICU. Bacterial colonization became evident in all preterm neonates aged 2 weeks and older. This is in contrast to our previous study of healthy full-term neonates. In that earlier work, the gut of healthy full-term neonates showed initial colonization with the normal resident enterobacterium *E. coli* as early as days 5 and 6 in the second half of the first week of their life in all healthy full-term neonate investigated subjects despite the delay in neonates born by cesarean section compared to vaginal birth (Al-Balawi and Morsy, 2021).

Carbapenem-resistant gram-negative bacteria, such as carbapenem-resistant Enterobacteriaceae, are considered multidrug-resistant bacteria. The occurrence of carbapenem-resistant enterobacterium *K. pneumoniae* in the stool of preterm neonates highlights the incubation and possibly the creation site of these multidrug-resistant strains, colonizing the gut of preterm neonates intravenously treated with antibiotics in NICUs. Moreover, we cannot rule out a generation of carbapenem-multidrug-resistant *E. coli* strains due to the relatively small sample size of critically ill preterm neonates eligible for enrollment. The multidrug-resistant bacteria may lead to virtually untreatable infections, where such pathogens might acquire resistance determinants for multiple antibiotic classes (Abat et al., 2018). Carbapenem-resistant enterobacteria infections represent an important therapeutic problem, as other effective therapeutic alternatives are limited (Gajdács et al., 2020). The carbapenem class of antibiotics is usually reserved for the treatment of infections caused by known or suspected multidrug-resistant bacteria (Elshamy and Aboshanab, 2020). The emergence of resistant bacteria to these last-resort effective therapeutic alternative antibiotics would be especially daunting in NICUs.

Concurrent with our results, a recent report from Serbia stated that carbapenem-resistant enterobacterium *K. pneumoniae* intestinal colonization was present in one-fourth of preterm neonates at discharge from a tertiary care center (Mijac et al., 2023). Our findings also align with emerging data from NICUs in Saudi Arabia and the broader Middle East, where neonatal populations remain particularly vulnerable to MDR Enterobacteriaceae colonization and infection. A high prevalence of carbapenem-resistant *K. pneumoniae* colonization was reported (Al-Abdely et al., 2021) among neonates in Saudi NICUs, underscoring the gut as a critical reservoir contributing to outbreaks and transmission in these settings. Similarly, MDR *K. pneumoniae* isolates were characterized from neonatal infections in Saudi hospitals (Alghoribi et al., 2020), revealing the presence of key carbapenemase genes responsible for resistance. These findings reinforce the importance of focusing surveillance efforts specifically on neonatal units, where immature immune systems and antibiotic exposure increase the susceptibility to colonization and subsequent infection.

The presence of *A. baumannii*, detected in 12.5% of the investigated preterm neonate subjects, indicates the ability of this non-Enterobacteriaceae bacterium to colonize the gut of preterm neonates along with enterobacteria. *A. baumannii* has been reported as responsible for outbreaks in NICUs and many infections in neonates (Zarrilli et al., 2018). This opportunistic pathogen has a high capacity for healthcare-associated infections (Wisplinghoff et al., 2004), particularly among immunocompromised individuals during prolonged hospital stays (Montefour et al., 2008; Howard et al., 2012), and hence might represent a risk for preterm neonates with an immature immune system. The colonization of preterm neonates’ gut by this bacterium should be monitored in NICU patients for treatment and to avoid this incubation site for such pathogens in NICUs. The study indicates that while *A. baumannii* strains colonizing preterm neonates’ gut do not exhibit carbapenem resistance like *E. coli*, *K. pneumoniae* poses a greater concern due to its established carbapenem resistance and potential for causing infections in NICUs. *Klebsiella pneumonia* represents an opportunistic healthcare-associated pathogen and is reported as a cause of various healthcare-associated infections other than bacterial pneumonia, which include bloodstream infections (Meatherall et al., 2009; Girometti et al., 2014; Durdu et al., 2016; Li and Huang, 2017), meningitis (Tang and Chen, 1994; Ku et al., 2017), and others (Abbas et al., 2024), where it is a major cause of neonatal sepsis (Mukherjee et al., 2021; Mukherjee et al., 2023). The ability of multidrug-resistant *K. pneumoniae* to colonize the gut of preterm neonates might indicate a high risk of infection for preterm neonates in NICUs.

Extended-spectrum beta-lactamase (ESBL)-producing bacteria which are considered resistant to many of the beta-lactam antibiotics (Husna et al., 2023) were detected in this study in four cases of *K. pneumoniae* and *E. coli.*

All strains of *K. pneumoniae*, including the carbapenem-multidrug-resistant and intermediate resistant strains, were sensitive to gentamicin and tigecycline. Despite the sensitivity of the carbapenem-multidrug-resistant and the intermediate resistant strains of *K. pneumoniae* to trimethoprim/sulfamethoxazole (SXT) and amikacin (AK), respectively, the action of these antibiotics was not broad against all other strains of *K. pneumoniae.* In contrast, the sensitivity to gentamicin and tigecycline was broad across all strains of *K. pneumoniae.* Thus, of all the tested antibiotics in this study, gentamicin and tigecycline may be applicable for treatment against all strains of *K. pneumoniae* in this study, including carbapenem-multidrug-resistant *K. pneumoniae.* The antibiotic resistance profiles are consistent with resistance trends documented in neonatal isolates from Saudi Arabian NICUs (Al-Agamy et al., 2009; Abdulhalim et al., 2023). Such patterns highlight ongoing challenges in managing neonatal infections and the critical need for effective antibiotic stewardship tailored to neonatal pharmacodynamics and microbiology.

In conclusion, this study highlights the important role of preterm neonates colonized with multidrug-resistant *K. pneumoniae*, particularly carbapenem-resistant strains, as reservoirs for healthcare-associated transmission in NICUs. These findings emphasize the need for the implementation of routine stool screening of high-risk neonates as part of NICU surveillance protocols, combined with enhanced infection control measures such as cohorting, isolation, and strict hygiene practices to limit the spread of pathogens. Longitudinal monitoring of the neonatal gut microbiota could provide early warning signs of MDR colonization before a clinical infection develops, thereby reducing cross-transmission risks and improving NICU patient outcomes.

This study has some limitations. The relatively small sample size reflects the restricted availability of critically ill preterm neonates eligible for enrollment, which limited the statistical power and generalizability of the findings. Nevertheless, the results in this study provide valuable preliminary insights into the colonization dynamics of multidrug-resistant *Klebsiella pneumoniae* in this high-risk group. Additionally, the partial availability of clinical records prevented the inclusion of certain relevant risk factors, such as gastrointestinal surgery, and restricted access to maternal infection history and antibiotic usage. Consequently, the analysis was primarily based on the neonates’ own clinical history and antibiotic exposure, which were consistently available. Future multicenter studies with larger cohorts and more comprehensive clinical data, including maternal parameters, are warranted to validate and expand upon the present findings.

A notable limitation of this study is the absence of investigation into novel biomarkers, such as specific resistance genes or virulence factors, that could distinguish MDR strains more precisely. Future research should incorporate molecular diagnostic tools, including PCR assays targeting resistance and virulence genes and whole-genome sequencing, to identify such biomarkers. These molecular insights would enable rapid and accurate detection and better epidemiological tracking of MDR *K. pneumoniae* in NICU environments. Furthermore, establishing baseline gut microbiota profiles in preterm neonates and comparing them with those of healthy full-term neonates would enhance the understanding of colonization dynamics and inform tailored prevention strategies.

Collectively, these combined phenotypic and molecular approaches have the potential to significantly improve NICU surveillance, infection control protocols, and ultimately neonatal health outcomes.

## Data Availability

The datasets presented in this study can be found in online repositories. The names of the repository/repositories and accession number(s) can be found in the article/**Supplementary Material**.
