# Supplementary material for: Colonization of the gut by Klebsiella pneumoniae and its multidrug-resistant strains is well marked in preterm neonates
Source: Front Cell Infect Microbiol. 2026 Apr 29;16:1762624. doi: 10.3389/fcimb.2026.1762624 (PMC13168211; doi:10.3389/fcimb.2026.1762624)
Supplement: Supplementary file 1 [file Table1.docx]

Supplementary Table S1: *In vitro* antibiotics sensitivity of various ***Klebsiella pneumoniae*** and *Klebsiella quasipneumoniae* (Enterobacteriaceae) strains and *Acinetobacter baumannii* (a non-Enterobacteriaceae bacterium) colonizing preterm neonates’ gut.

| Bacterial strains | ESBL | | Antibiotics | | | | | | | | | | | | | | | |
| --- | --- | --- | --- | --- | --- | --- | --- | --- | --- | --- | --- | --- | --- | --- | --- | --- | --- | --- |
|  |  | AMP | | | Amox | | Tzp | | CF | | FOX | | CAZ | | CRO | | FEP | |
|  |  | MIC | | Intpr | MIC | Intpr | MIC | Intpr | MIC | Intpr | MIC | Intpr | MIC | Intpr | MIC | Intpr | MIC | Intpr |
| ***Klebsiella pneumoniae*** EFTU101 | P0S | >=32 | | R | 4 | S | <=4 | S | >=64 | R | <=4 | S | 8 | R | >=64 | R | >=64 | R |
| ***Klebsiella pneumoniae*** EFTU102 | NEG | >=32 | | R | <=2 | S | <=4 | S | <=2 | S | <=4 | S | <=1 | S | <=1 | S | <=1 | S |
| *Klebsiella pneumoniae* EFTU103 | NEG | >=32 | | R | <=2 | S | <=4 | S | <=2 | S | <=4 | S | <=1 | S | <=1 | S | <=1 | S |
| *Klebsiella pneumoniae* EFTU104 | P0S | >=32 | | R | 16 | i | 32 | i | >=64 | R | 16 | R | >=64 | R | >=64 | R | >=64 | R |
| *Klebsiella pneumoniae* EFTU105 | P0S | >=32 | | R | 8 | S | <=4 | S | >=64 | R | <=4 | S | 8 | R | >=64 | R | 2 | R |
| *Klebsiella pneumoniae* EFTU3 | NEG | >=32 | | R | 4 | S | <=4 | S | 16 | I | <=4 | S | <=1 | S | <=1 | S | <=1 | S |
| *Klebsiella pneumoniae* EFTU110 | NEG | >=32 | | R | >=32 | R | ≥128 | R | >=64 | R | >=64 | R | 16 | R | >=64 | R | >=64 | R |
| *Acinetobacter baumannii* EFTU111 | NM | NM | | - | NM | - | <=4 | S | NM | - | NM | - | 4 | S | 16 | R | 2 | S |
| *Klebsiella pneumoniae* EFTU113 | P0S | >=32 | | R | >=32 | R | 3 | R | >=64 | R | <=4 | R | >=64 | R | >=64 | R | >=64 | R |
| *Acinetobacter baumannii* EFTU114 | NM | NM | | - | NM | - | <=4 | S | NM | - | NM | - | 4 | S | 8 | R | 2 | S |
| *Klebsiella quasipneumoniae* EFTU115 | NM | NM | | - | NM | - | <=4 | S | NM | - | NM | - | 4 | S | 8 | R | 2 | S |
| *Klebsiella pneumoniae* EFTU116 | NEG | >=32 | | R | >=32 | R | 8 | S | >=64 | R | >=64 | R | 4 | S | <=1 | S | <=1 | S |

Supplementary Table S1 continued:

| Bacterial strains | Antibiotics | | | | | | | | | | | | | | | |
| --- | --- | --- | --- | --- | --- | --- | --- | --- | --- | --- | --- | --- | --- | --- | --- | --- |
|  | IMI | | MERO | | AK | | GM | | CIP | | TGC | | FT | | SXT | |
|  | MIC | Intpr | MIC | Intpr | MIC | Intpr | MIC | Intpr | MIC | Intpr | MIC | Intpr | MIC | Intpr | MIC | Intpr |
| ***Klebsiella pneumoniae*** EFTU101 | <=0,25 | S | <=0,25 | S | <=2 | S | <=1 | S | 0, 5 | S | 1 | S | 64 | I | 320 | R |
| ***Klebsiella pneumoniae*** EFTU102 | <=0,25 | S | <=0,25 | S | <=2 | S | <=1 | S | <=0, 25 | S | =<0,5 | S | 32 | S | 20 | S |
| *Klebsiella pneumoniae* EFTU103 | <=0,25 | S | <=0,25 | S | <=2 | S | <=1 | S | <=0, 25 | S | =<0,5 | S | 32 | S | 20 | S |
| *Klebsiella pneumoniae* EFTU104 | <=0,25 | S | <=0,25 | S | <=2 | S | <=1 | S | 1 | S | 1 | S | 128 | R | >=320 | R |
| *Klebsiella pneumoniae* EFTU105 | <=0,25 | S | <=0,25 | S | <=2 | S | <=1 | S | 1 | S | 2 | S | 64 | I | >=320 | R |
| *Klebsiella pneumoniae* EFTU3 | <=0,25 | S | <=0,25 | S | <=2 | S | <=1 | S | <=0,25 | S | <=0,5 | S | <=16 | S | <=20 | S |
| *Klebsiella pneumoniae* EFTU110 | >=16 | R | >=16 | R | ≥64 | R | <=1 | S | >=4 | R | 2 | S | 256 | R | 40 | S |
| *Acinetobacter baumannii* EFTU111 | <=0,25 | S | <=0,25 | S | NM | - | <=1 | S | <=0,25 | S | <=0,5 | S | NM | - | <=20 | S |
| *Klebsiella pneumoniae* EFTU113 | 2 | I | 2 | I | 4 | S | 2 | S | 2 | R | 2 | S | 128 | R | <=320 | R |
| *Acinetobacter baumannii* EFTU114 | <=0,25 | S | <=0,25 | S | NM | - | <=1 | S | <=0,25 | S | <=0,5 | S | NM | - | <=20 | S |
| *Klebsiella quasipneumoniae* EFTU115 | <=0,25 | S | <=0,25 | S | NM | - | <=1 | S | <=0,25 | S | <=0,5 | S | NM | - | <=20 | S |
| *Klebsiella pneumoniae* EFTU116 | <=0,25 | S | <=0,25 | S | <=2 | S | <=1 | S | 1 | S | 2 | S | 128 | R | 320 | R |

Interpretation, Intpr; Resistant, R; Sensitive, S; Intermediate, I; Minimum Inhibitory Concentration, MIC (µg/ml); Negative, Neg; Positive, Pos. Extended-spectrum beta-lactamases, ESBL; NM, Not measured; Ampicillin/Sulbactam, AMP; Amoxicillin/Clavulanic Acid, AMOX; Piperacillin/Tazobactam, TZP; Cefalotin, CF; Cefoxitin, FOX; Ceftazidime, CAZ; Ceftriaxone, CRO; Cefepime, FEP; Imipenem, IMI; Meropenem, MERO; Amikacin, AK; Gentamicin, GM; Ciprofloxacin, CIP; Tigecycline, TGC; Nitrofurantoin, FT; Trimethoprim/Sulfamethoxazole, SXT.
