# Supplementary material for: Colonization of the gut by Klebsiella pneumoniae and its multidrug-resistant strains is well marked in preterm neonates
Source: Front Cell Infect Microbiol. 2026 Apr 29;16:1762624. doi: 10.3389/fcimb.2026.1762624 (PMC13168211; doi:10.3389/fcimb.2026.1762624)
Supplement: Supplementary file 2 [file Table2.docx]

Supplementary Table S2: *In vitro* antibiotics sensitivity of various *Escherichia coli* strains colonizing preterm neonates’ gut.

| Strains | ESBL | Antibiotics | | | | | | | | | | | | | | | |
| --- | --- | --- | --- | --- | --- | --- | --- | --- | --- | --- | --- | --- | --- | --- | --- | --- | --- |
|  |  | AMP | | Amox | | Tzp | | CF | | FOX | | CAZ | | CRO | | FEP | |
|  |  | MIC | Intpr | MIC | Intpr | MIC | Intpr | MIC | Intpr | MIC | Intpr | MIC | Intpr | MIC | Intpr | MIC | Intpr |
| *E. coli* EFTU 1 | NEG | >=32 | R | 4 | S | <=4 | S | 16 | I | <=4 | S | <=1 | S | <=1 | S | <=1 | S |
| *E. coli* EFTU 2 | POS | >=32 | R | 4 | S | <=4 | S | >=64 | R | <=4 | S | 4 | R | <=64 | R | 2 | R |
| *E. coli* EFTU 4 | NEG | >=32 | R | 4 | S | <=4 | S | 16 | I | <=4 | S | <=1 | S | <=1 | S | <=1 | S |
| *E. coli* ETTU5 | NEG | <=2 | S | <=2 | S | <=4 | S | 4 | S | <=4 | S | <=1 | S | <=1 | S | <=1 | S |
| *E. coli* EFTU6 | NEG | >=32 | R | 16 | I | <=4 | S | 16 | I | <=4 | S | <=1 | S | <=1 | S | <=1 | S |
| *E. coli* EFTU7 | P0S | >=32 | R | 4 | S | <=4 | S | >=64 | R | <=4 | S | <=1 | R | >=64 | R | <=1 | R |
| *E. coli* EFTU8 | P0S | >=32 | R | 4 | S | <=4 | S | >=64 | R | <=4 | S | 16 | R | >=64 | R | 8 | R |
| *E. coli* EFTU9 | P0S | >=32 | R | 4 | S | <=4 | S | >=64 | R | <=4 | S | 16 | R | >=64 | R | >=64 | R |

Supplementary Table S2 continued:

| Strains | Antibiotics | | | | | | | | | | | | | | | |
| --- | --- | --- | --- | --- | --- | --- | --- | --- | --- | --- | --- | --- | --- | --- | --- | --- |
|  | IMI | | MERO | | AK | | GM | | CIP | | TGC | | FT | | SXT | |
|  | MIC | Intpr | MIC | Intpr | MIC | Intpr | MIC | Intpr | MIC | Intpr | MIC | Intpr | MIC | Intpr | MIC | Intpr |
| *E. coli* EFTU 1 | <=0,25 | S | <=0,25 | S | <=2 | S | <=1 | S | <=0,25 | S | <=0,5 | S | <=16 | S | <=20 | S |
| *E. coli* EFTU 2 | <=0,25 | S | <=0,25 | S | <=2 | S | <=1 | S | <=0,25 | S | <=0,5 | S | <=16 | S | <=20 | S |
| *E. coli* EFTU 4 | <=0,25 | S | <=0,25 | S | <=2 | S | <=1 | S | <=0,25 | S | <=0,5 | S | <=16 | S | <=20 | S |
| *E. coli* ETTU5 | <=0,25 | S | <=0,25 | S | <=2 | S | <=1 | S | <=0,25 | S | <=0, 5 | S | <=16 | S | <=20 | S |
| *E. coli* EFTU6 | <=0,25 | S | <=0,25 | S | <=2 | S | <=1 | S | <=0,25 | S | <=0,5 | S | <=16 | S | >=320 | R |
| *E. coli* EFTU7 | <=0,25 | S | <=0,25 | S | <=2 | S | <=1 | S | <=0,25 | S | <=0, 5 | S | <=16 | S | <=20 | S |
| *E. coli* EFTU8 | <=0,25 | S | <=0,25 | S | <=2 | S | <=1 | S | 0, 5 | S | <=0, 5 | S | <=16 | S | <=20 | S |
| *E. coli* EFTU9 | <=0,25 | S | <=0,25 | S | <=2 | S | <=1 | S | 0, 5 | S | <=0, 5 | S | <=16 | S | <=20 | S |

Interpretation, Intpr; Resistant, R; Sensitive, S; Intermediate, I; Minimum Inhibitory Concentration, MIC (µg/ml); Negative, Neg; Positive, Pos. Extended-spectrum beta-lactamases, ESBL; NM, Not measured; Ampicillin/Sulbactam, AMP; Amoxicillin/Clavulanic Acid, AMOX; Piperacillin/Tazobactam, TZP; Cefalotin, CF; Cefoxitin, FOX; Ceftazidime, CAZ; Ceftriaxone, CRO; Cefepime, FEP; Imipenem, IMI; Meropenem, MERO; Amikacin, AK; Gentamicin, GM; Ciprofloxacin, CIP; Tigecycline, TGC; Nitrofurantoin, FT; Trimethoprim/Sulfamethoxazole, SXT.
